# Beyond platelet response: toward an immune remission framework in immune thrombocytopenia

**DOI:** 10.3389/fimmu.2026.1974216

**Published:** 2026-10-20

**Authors:** Zhenglun Pan

**Affiliations:** Department of Rheumatology and Immunology, Qilu Hospital (Qingdao), Cheeloo College of Medicine, Shandong University, Qingdao, China

**Keywords:** clinical remission, immune dysregulation, immune homeostasis, immune remission, immune thrombocytopenia, multidimensional immune architecture, platelet response, translational immunology

## Abstract

Platelet response and clinical remission remain central measures of clinical control in immune thrombocytopenia (ITP), but they do not establish whether disease-relevant immune activity has durably receded. We propose immune remission as a provisional, multidimensional research construct: a sustained reduction in disease-relevant immune activity across more than one mechanistic domain, demonstrated longitudinally beyond platelet recovery and treatment-related effects, and evaluated against durable clinical control or relapse. The framework comprises six interconnected analytical domains: B-cell regulation, T-cell regulation, the Fc/antibody interface, the bone marrow immune niche, complement regulation, and innate immune activation. These domains are pragmatic modules rather than independent pathways, and they differ in causal position, measurability, and evidence maturity. The framework does not require normalization of every immune measure to a healthy-control range or elimination of all autoreactive lymphocytes. It preserves platelet response, bleeding risk, and treatment burden as clinical anchors and does not currently support routine immune testing, prediction, treatment extension, or withdrawal decisions. Operationalization requires prespecified assays and time points, longitudinal within-patient analysis, separation of disease change from treatment effects, external validation, and demonstration of added clinical value. Immune remission is therefore presented as a testable research framework, not a validated clinical endpoint.

## 1 Introduction

Immune thrombocytopenia (ITP) is an acquired autoimmune bleeding disorder characterized by thrombocytopenia caused by accelerated platelet destruction and impaired platelet production. Its clinical expression is heterogeneous: bleeding ranges from absent or mild mucocutaneous manifestations to uncommon major hemorrhage, while disease may be newly diagnosed, persistent, or chronic. Remission may occur spontaneously or after treatment, and treatment-free response represents an important clinical state. Primary ITP is diagnosed when no underlying disorder is identified; secondary ITP occurs in association with conditions such as systemic autoimmune disease, infection, immunodeficiency, lymphoproliferative disease, or selected drugs. The present framework is directed primarily to primary ITP, while recognizing that diagnostic classification can evolve during longitudinal follow-up (1–6).

Contemporary pathophysiology extends beyond a single autoantibody-clearance pathway. Platelet autoantibodies can promote Fcγ receptor-mediated phagocytosis, complement activation, desialylation, apoptosis, and impaired megakaryocyte maturation or platelet production. Cytotoxic T cells may directly injure platelets and megakaryocytes, while abnormalities of regulatory, helper, follicular helper, and follicular regulatory T cells can sustain loss of tolerance and B-cell help. Autoreactive B cells and plasma cells, altered Fc-effector function, innate immune activation, and changes within the bone marrow immune niche further contribute to heterogeneity among patients and over time (3–8). The clinical tractability of this biology is further supported by therapies that reduce FcRn-mediated IgG recycling or inhibit Fc receptor-associated effector signaling, although treatment response alone cannot identify the dominant mechanism in an individual patient (4).

Platelet count recovery, bleeding control, treatment response, toxicity, treatment-free response, and patient-reported outcomes nevertheless remain the practical anchors of ITP assessment (1–3). This platelet-centered framework is clinically appropriate because bleeding is the immediate danger and unnecessary long-term immunosuppression should be avoided. A related biological question remains unresolved: platelet recovery does not by itself show whether disease-relevant immune activity has durably receded, yet persistence of an immune abnormality does not necessarily indicate clinically meaningful disease.

The unmet need is therefore not a replacement for clinical remission, but a research framework connecting immune-remission language to an operational definition, prespecified measurement, longitudinal behavior, and clinical meaning. Observations differ by population, anatomical compartment, treatment, assay, and evidence maturity and cannot be combined automatically into a panel, threshold, or prediction tool (7–13). **Figure 1** summarizes the distinction between clinical and immune-state assessment; **Figure 2** presents the interconnected six-domain architecture; and **Tables 1**, **2** outline its conceptual and validation structure.

**Figure 1:**
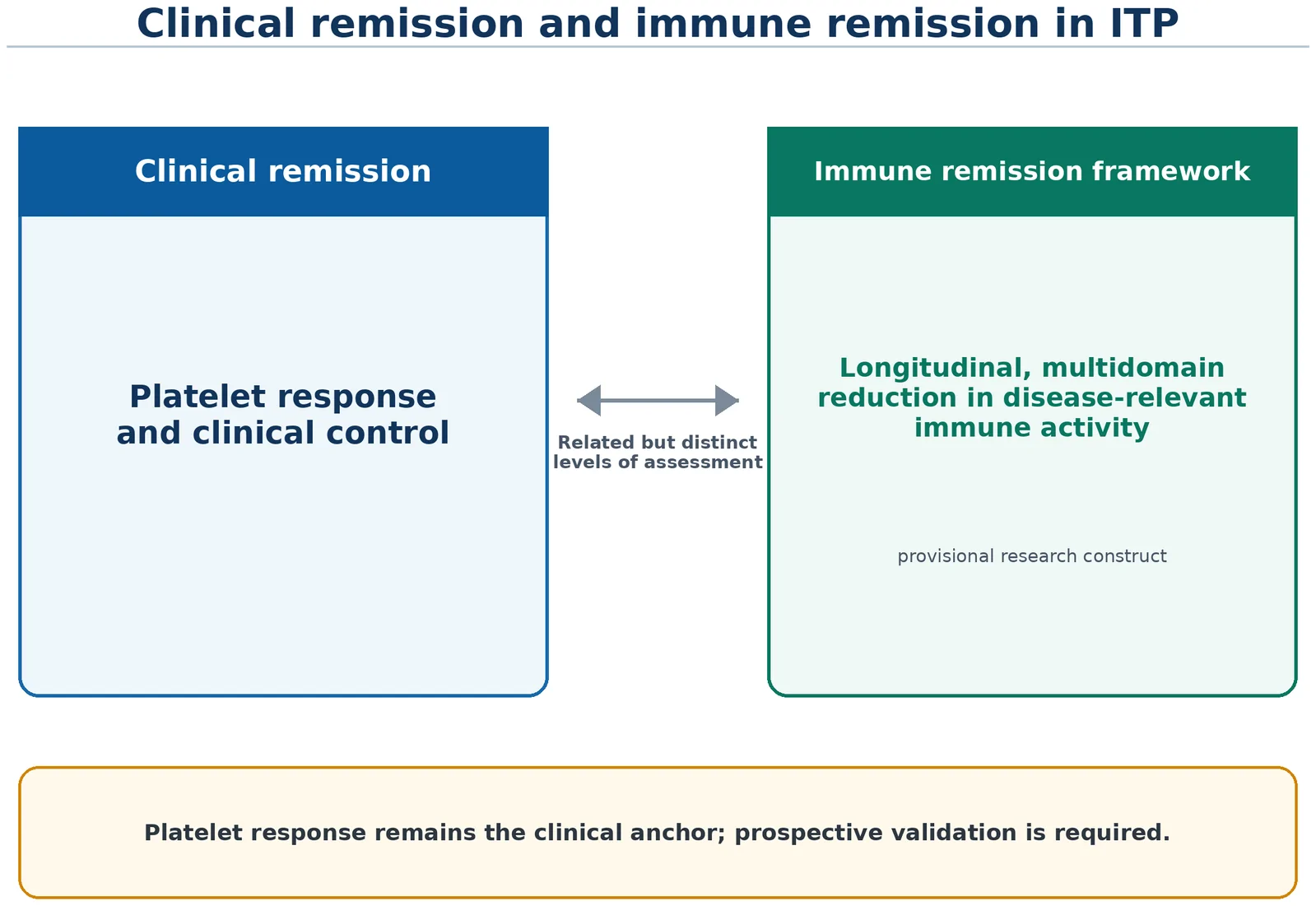
Clinical remission and immune remission as related levels of assessment in ITP. Clinical remission is anchored to platelet response and clinical control. Immune remission is a provisional multidimensional research construct requiring longitudinal demonstration beyond platelet recovery and treatment effects. The figure does not imply that every clinical remission retains immune abnormality, that healthy-control values establish tolerance, or that immune remission is a validated clinical endpoint.

**Figure 2:**
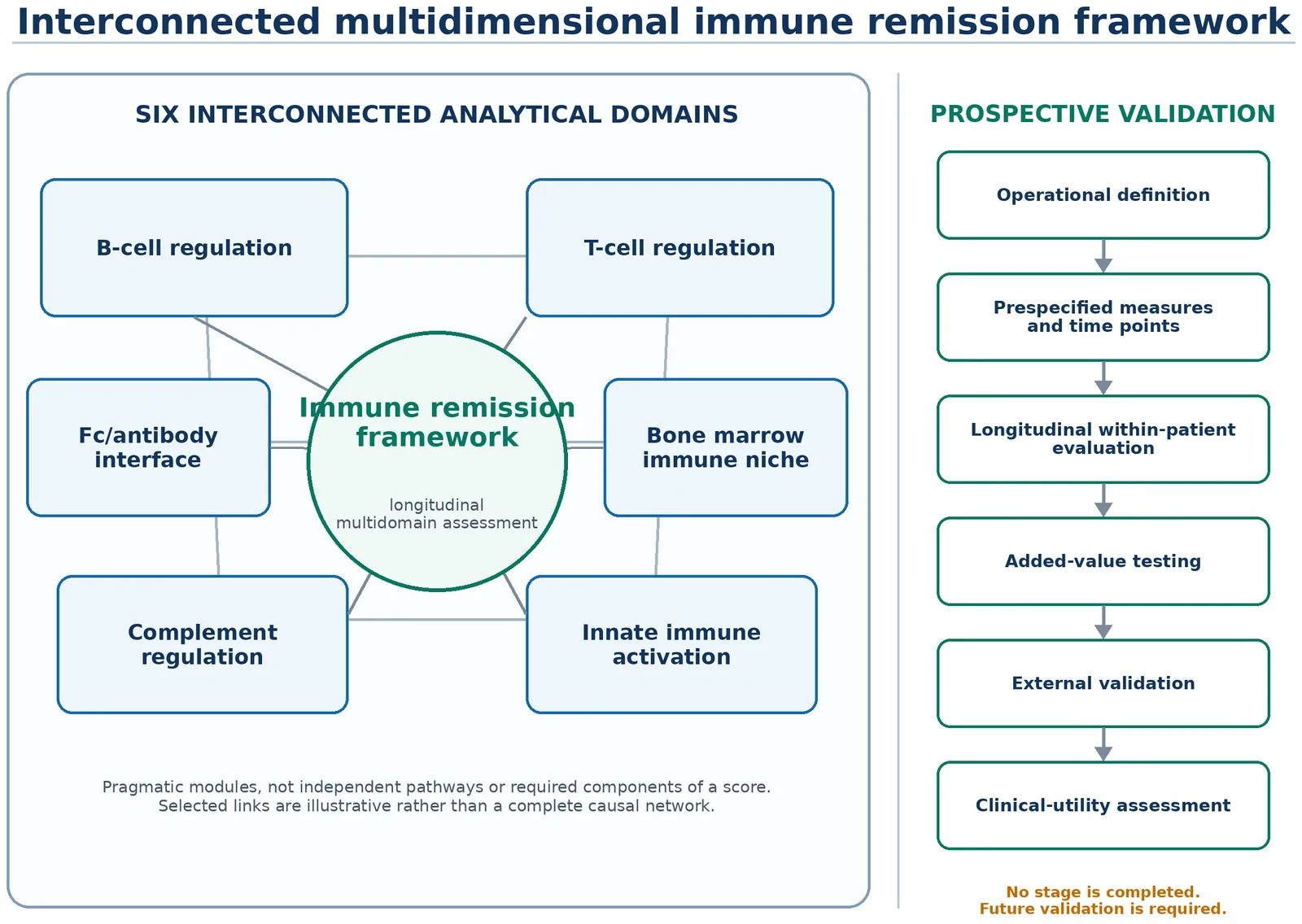
Multidimensional immune remission architecture. The framework organizes six interconnected analytical domains: B-cell regulation, T-cell regulation, the Fc/antibody interface, the bone marrow immune niche, complement regulation, and innate immune activation. Connecting lines indicate selected biologically plausible cross-domain relationships, not a complete causal network. Visual placement does not imply equal causal importance, measurability, or evidence maturity. The staged pathway on the right summarizes prospective operationalization and validation; no stage is presented as completed.

**Table 1:** Remission logic framework.

Conceptual level	Immune thrombocytopenia	Systemic lupus erythematosus	Implication for ITP
Clinical goal and routine endpoint	Platelet safety, bleeding control, treatment response, treatment toxicity, and patient-reported outcomes	Control of clinical activity and prevention of organ damage	ITP endpoints remain platelet- and bleeding-centered
Current remission framework	Hematologic response, sustained response, treatment-free response, and reduced rescue therapy	Remission or low disease activity incorporates clinical activity and treatment exposure	The ITP framework is clinically useful but does not define immune remission
Treatment-duration and tapering decisions	Guided by platelet stability, bleeding risk, relapse, toxicity, patient preference, and monitoring feasibility	Guided by clinical activity, organ risk, flares, treatment toxicity, and long-term damage prevention	SLE is not a therapeutic template for ITP; ITP tapering remains disease-specific
Immune activity monitoring	No routine immune measure defines immune remission	Selected serological variables contribute to disease assessment	Candidate immune measures in ITP require prospective evaluation
Clinical remission-immune activity discordance	Not formally defined; treatment-free response does not automatically establish immune restoration	Serologically active clinically quiescent SLE illustrates possible discordance	Clinical and immune assessment may address related but distinct questions
Implication for immune remission framework in ITP	Platelet remission is necessary for clinical control but may not answer the broader immune-state question	Clinical remission and immune activity can be dissociated	SLE provides a conceptual precedent, not a transferred endpoint or treatment strategy

SLE is included as a limited methodological precedent and not as a biological or therapeutic template for ITP (1–3, 14–17, 41, 45, 46).

**Table 2:** Candidate measurements and endpoints for developing and validating an immune remission framework in ITP.

Category	Candidate measurements or endpoints	Primary interpretation	Research role and priority	Key limitation
Mechanistic measurements	Anti-GPIIb/IIIa, anti-GPIb/IX, anti-GPIa/IIa, and anti-GPVI antibodies	Platelet-directed humoral recognition and candidate effector routing	Exploratory pathway characterization; moderate feasibility	Assay heterogeneity; incomplete sensitivity; no remission threshold
Mechanistic measurements	Fc quality and function, platelet-bound	Antibody persistence, Fc/FcγR competence,	Exploratory mechanism separation;	Specialized assays; individual mechanism
	complement, desialylation or apoptosis assays	amplification, and non-phagocytic injury	limited feasibility	attribution remains uncertain
Mechanistic measurements	Megakaryocyte injury, marrow immune or stromal programs, immature platelet fraction, serum TPO	Production-side injury and compensatory thrombopoiesis	Selected cohorts; integrate destruction and impaired production	Marrow sampling is invasive; peripheral surrogates are unvalidated
Immune-state measurements	B-cell subsets, plasmablasts, antigen-specific clones, BAFF/APRIL	B-lineage activation, persistence, recruitment, and survival	Priority discovery domain; longitudinal trajectories	Blood may not represent spleen or marrow; assays are not standardized
Immune-state measurements	Treg function, Tfh/Tfr, Th1/Th2, Th17, and cytotoxic T-cell signatures	Regulation, B-cell help, inflammatory polarization, and cellular injury	Priority discovery domain; longitudinal trajectories	Subset frequency may not reflect function; compartment dependence
Immune-state measurements	Monocyte/macrophage states, neutrophil programs, cytokine or interferon signatures	Innate activation and inflammatory context	Exploratory multidomain concordance; moderate feasibility	Assay variability; peripheral and tissue states may diverge
Immune-state measurements	ANA, anti-dsDNA, anti-Sm, SSA/SSB, antiphospholipid antibodies, C3/C4	Contextual systemic-autoimmunity features	Subgroup context only; not a core endpoint	Not direct measures of ITP activity or immune remission
Clinical anchors	Platelet response, bleeding, rescue therapy, corticosteroid dependence, exposure and toxicity	Current disease control and treatment context	Mandatory anchor for interpreting immune trajectories	Do not establish immune restoration
Clinical anchors	Treatment-free remission, TPO-RA taper outcome, relapse after withdrawal	Durability of control without active therapy	Mandatory longitudinal clinical anchor	Affected by mechanism, treatment, and follow-up duration
Clinical anchors	Fatigue, bleeding anxiety, treatment burden, health-related quality of life	Patient-experienced disease impact	Recommended contextual outcomes	Subjective and multifactorial
Validation endpoints	Within-patient change at prespecified time points	Temporal stability beyond baseline heterogeneity	First validation step	Requires standardized timing and adequate follow-up
Validation endpoints	Added prediction beyond clinical variables	Incremental information for sustained control or relapse	After derivation; prespecified analysis	Risk of overfitting
Validation endpoints	Independent multicenter replication and assay transferability	Generalizability and measurement robustness	Required before clinical claims	Batch, platform, and population effects
Validation endpoints	Decision impact, benefit, burden, and cost	Clinical utility	Final stage only	Not appropriate before validity is established

Candidate measures are grouped by their role in inference rather than treated as components of a score. Mechanistic and immune-state measures require prespecified assays and timing; clinical anchors remain the basis of care; validation endpoints test reproducibility and added value. None is a current treatment indication (7–13, 42–44).

## 2 A multidimensional immune remission framework

For research purposes, we provisionally define immune remission in ITP as a sustained, multidomain reduction in disease-relevant immune activity, demonstrated longitudinally beyond platelet recovery and treatment-related effects, and evaluated for association with maintained clinical control or reduced relapse risk. This definition is deliberately testable but not yet validated. It does not require normalization of every immune measurement to the healthy-control range, elimination of all autoreactive lymphocytes, or concordant change in all six domains.

Immune homeostasis and immune remission are therefore not interchangeable. Immune homeostasis denotes a broader biological balance that cannot currently be captured by a single reference range. Immune remission is the narrower investigational construct used to test whether disease-relevant immune trajectories have changed in a sustained and clinically informative manner. Persistent autoreactive memory may be biologically relevant without being sufficient to define active disease; conversely, values resembling healthy controls do not establish restored tolerance. SLE offers a limited methodological precedent for separating clinical state, treatment exposure, and immune activity, but it is not a biological or therapeutic template for ITP (14–17).

## 3 Multidimensional immune architecture

The six domains were selected pragmatically to cover three complementary levels of ITP biology: adaptive immune regulation (B- and T-cell regulation), effector injury and amplification (the Fc/antibody interface, complement, and innate immune activation), and the tissue context of platelet production (the bone marrow immune niche). They are analytically distinguishable but biologically interconnected modules, not independent pathways, an exhaustive taxonomy, or six required components of a future score. B-cell and T-cell regulation shape antibody generation; antibody properties influence Fc- and complement-mediated injury; innate cells execute or modify clearance; and marrow-resident adaptive and innate populations interact with megakaryocytes and stromal cells.

This organization separates biological coverage from evidentiary equivalence. The contribution and measurability of each domain vary among patients, compartments, treatments, and time points. An abnormality may persist after platelet response, normalize with recovery, or reflect treatment exposure rather than durable biological change. **Figure 2** therefore depicts selected cross-domain relationships while avoiding a complete causal network. Determining whether coordinated or discordant trajectories carry clinical.

meaning requires prespecified longitudinal studies rather than inference from a single accessible blood measurement.

### 3.1 B-cell regulation

Platelet-directed autoantibody production provides a biological rationale for examining autoreactive B-lineage cells beyond circulating antibody concentrations. Antigen-specific studies have identified splenic anti-αIIbβ3 plasma cells after rituximab, including residual cells with a long-lived plasma-cell program (18). In relapse after an initial rituximab response, splenic repertoire and single-cell analyses have identified contributions from both treatment-resistant memory clones and newly engaged naïve B cells (19). These observations suggest that B-lineage activity may be sustained through persistence, renewed recruitment, or both.

Recent evidence extends this mechanism across anatomical compartments and developmental stages. High-affinity autoreactive IgG-secreting cells have been identified across spleen, marrow, and blood, with heterogeneous persistence during selected plasma-cell-directed treatment observations (20). Paired single-cell RNA and B-cell receptor sequencing, antibody cloning, and experimental validation have also suggested insufficient receptor editing and increased autoreactive or polyreactive naïve B cells in ITP (21). Together with BAFF/APRIL-associated survival biology and heterogeneous responses to B-cell depletion (22), these findings suggest that B-cell dysregulation may involve the generation of autoreactive cells, memory-clone persistence, plasma-cell survival, and renewed antigen-driven recruitment.

This layered biology provides a mechanistically supported explanation for asynchronous platelet and B-lineage recovery. Platelet counts may rise when clearance is interrupted, thrombopoiesis increases, or immune activity is suppressed before memory or plasma-cell compartments have changed substantially. Conversely, persistence of a clonotype or antigen-reactive cell does not establish continued secretion, sufficient effector activity, or relapse. B-cell findings may therefore help distinguish loss of autoreactive activity from compensated persistence or broader immune reconfiguration, but they do not define immune remission or support clinical selection.

The opposite pattern is also possible: circulating B-cell numbers may reconstitute without recreating the antigen-specific architecture that contributed to active disease. Numerical recovery, repertoire recovery, and restoration of tolerance are therefore separate constructs. Their relationship may depend on the treatment used, the tissue reservoir sampled, and whether autoreactive output is measured directly rather than inferred from total-cell frequencies.

Future work should distinguish antigen-specific secretion from clonotype persistence and sample treatment, platelet response, sustained treatment-free follow-up, and relapse. Blood measures require direct concordance testing against relevant tissue compartments. Repertoire and receptor-editing analyses need prespecified pipelines and independent replication. Whether B-lineage trajectories add reproducible information beyond platelet and clinical outcomes requires prospective validation.

### 3.2 T-cell regulation

T-cell regulation links loss of tolerance, B-cell help, inflammatory polarization, and antibody-independent effector injury. Studies in ITP describe Th1-skewed responses, disturbance of Th1/Th2 balance, expansion or activation of Th17 cells, and quantitative or functional defects in regulatory T cells. Abnormal Tfh activity and altered Tfh/Tfr balance may promote autoreactive B-cell selection and antibody production, whereas platelet-reactive cytotoxic CD8+ T cells can injure platelets and megakaryocytes directly. These findings vary by cohort, assay, compartment, and treatment and should not be assumed to coexist in every patient (7, 10, 23).

Paired blood and marrow studies indicate that T-cell distributions are compartment dependent, and high-dimensional marrow profiling suggests altered co-stimulatory and co-inhibitory states involving T cells and antigen-presenting populations (23, 24). A small prospective study identified abnormalities in Treg, NKT, CD8, and CD4/CD8 measures, but the measured subsets did not distinguish short-term treatment response (25). Recent synthesis has further proposed that incomplete exhaustion-associated regulatory programming, including checkpoint, epigenetic, and immunometabolic features, may help sustain autoreactive activity (26). This remains an emerging model rather than an established remission marker.

The central longitudinal question is not whether one peripheral ratio returns to normal, but whether regulatory function, helper activity, cytotoxic potential, repertoire, and memory change in a coordinated and durable way. Platelet recovery could occur while autoreactive T-cell memory persists; however, persistence alone does not establish clinically meaningful disease or predict relapse. Conversely, numerical normalization may coexist with defective suppressive function or tissue-restricted activity. Candidate T-cell measures should therefore be interpreted as trajectories and linked to functional assays, treatment exposure, and clinical follow-up.

T-cell biology also illustrates why the six domains cannot be treated as parallel independent processes. Tfh/Tfr balance and Treg function influence B-cell selection and antibody output; cytotoxic T cells may damage platelets or megakaryocytes without antibody; and innate antigen-presenting cells shape helper and regulatory programs. The same subset shift could represent a driver, a compensatory response, a pharmacological effect, or a consequence of reduced antigen and inflammatory exposure.

Prospective studies should prioritize reproducible functional measures alongside selected phenotypic, repertoire, and epigenetic or metabolic assessments; distinguish blood from marrow; and record treatment exposure, platelet response, treatment withdrawal, and relapse. The incremental value of any T-cell trajectory must be tested beyond these clinical variables and replicated independently.

### 3.3 Fc/antibody interface

The Fc/antibody interface links platelet recognition to clearance and other forms of injury. Antibodies against GPIIb/IIIa and GPIb/IX establish glycoprotein-specific recognition, but antigen specificity may route injury differently. Purified GPIb/IX-specific IgG has induced complement-independent platelet apoptosis ex vivo, illustrating an antibody effect not reducible to Fc-dependent phagocytosis (27). Human spleen studies also identify altered macrophage activation, phagocytic context, and inhibitory FCGRIIB expression (28, 29). These findings suggest heterogeneity at both recognition and effector-cell levels.

Therapeutic studies provide human pathway-perturbation evidence across distinct levels of the Fc/antibody axis. Inhibition of SYK with fostamatinib targets signaling downstream of activating Fcγ receptors and can reduce antibody-dependent phagocytic clearance (30). FcRn inhibition reduces IgG recycling and can alter platelet trajectories in some adults with persistent or chronic ITP, as investigated with efgartigimod and rozanolixizumab (31, 32). BTK inhibition with rilzabrutinib may affect both B-cell and Fc receptor-associated pathways, making its clinical effects biologically informative but not specific to a single mechanism (33). Together, these interventions support the tractability of the Fc/antibody axis while also showing why a platelet response to pathway perturbation cannot, by itself, identify the dominant mechanism in an individual patient. In parallel, one adult cohort showed reduced global IgG sialylation and increased agalactosylation, suggesting that Fc glycan quality may add information distinct from antibody amount or specificity (34). The glycan finding is global rather than platelet-antigen specific and remains cross-sectional.

Antibody-mediated ITP may therefore involve several linked but non-equivalent variables: source, specificity, amount and affinity, Fc quality, FcRn-dependent persistence, Fc-receptor balance, and macrophage competence. Interrupting IgG persistence or immune-effector signaling could change platelet counts before autoreactive memory or plasma-cell sources are removed. Conversely, antibody detection, a glycan pattern, an ex-vivo effect, or response to pathway perturbation does not establish the dominant mechanism in an individual.

The interface also prevents a false antibody-positive/antibody-negative dichotomy. Antibody-mediated injury may proceed through phagocytosis, apoptosis, desialylation, or complement-linked routes, whereas cellular injury and impaired production may contribute when conventional antibody assays are negative. Different routes could coexist or shift over time, so absence of one measured effector function does not establish broader immune restoration.

An immune-state assessment would need to separate antibody source from circulating antibody, Fc quality, downstream effector competence, and durability after treatment withdrawal. Prospective studies should integrate antigen-specific and functional measures across platelet response and treatment-free follow-up while retaining antibody-independent destruction and impaired production as alternatives. This interface may help explain asynchronous recovery, but any clinical application requires prospective validation and cannot be inferred from pathway response alone.

### 3.4 Bone marrow immune niche

The bone marrow provides the production-side and tissue context of ITP. Earlier human studies described altered megakaryocyte vascular localization, CXCL12/CXCR4 and stromal features, and mesenchymal stromal-cell injury (35). More recent marrow single-cell RNA sequencing identified altered progenitor programs and megakaryocytic trajectories; flow and culture experiments supported impaired megakaryocyte differentiation, including a bounded effect of ITP pre-B cells in coculture (36). High-dimensional proteomic profiling has independently suggested altered marrow immune-regulatory states (24).

Direct spatial and functional evidence further extends this niche beyond progenitor-intrinsic abnormalities. Human single-cell and spatial transcriptomics, primary-cell experiments, and mouse models have linked neutrophil-derived S100A8/A9 signaling to impaired megakaryocyte maturation (37). Functional models support biological plausibility, although they do not establish the dominant human mechanism or its longitudinal persistence. CSF1R–RUNX1 signaling represents another emerging megakaryocyte axis, but its discovery base and preclinical emphasis require replication (38).

These findings suggest that marrow should be viewed as an immune-production niche in which progenitors, megakaryocytes, stromal cells, B-lineage cells, T cells, monocytes, and neutrophils interact. Platelet production could improve through thrombopoietic stimulation even if immune or stromal abnormalities remain, but this discordance has not been established longitudinally. Conversely, peripheral immune control may reduce marrow inflammation and permit later tissue repair. Platelet and tissue recovery may therefore be concordant, lagging, or treatment dependent.

Cause and consequence are especially difficult to separate in marrow. Severe thrombocytopenia, increased platelet demand, inflammation, and prior therapy may themselves reshape progenitor and stromal states. Single-cell trajectories and inferred cell communication reveal candidate organization, but they do not establish temporal direction. Functional experiments strengthen plausibility, yet the relevance of an experimental rescue to spontaneous or treatment-associated human recovery remains uncertain.

Repeated marrow sampling is invasive, and peripheral measures cannot be assumed to represent tissue state. Longitudinal studies should define treatment and clinical state at sampling, replicate single-cell and spatial findings, and validate candidate interactions using human systems. They should also test whether accessible measures retain information about progenitor, megakaryocyte, stromal, or inflammatory programs. A marrow recovery trajectory may contribute to immune-state interpretation, but its relationship to durable immune remission requires prospective validation.

### 3.5 Complement regulation

Complement may provide a heterogeneous amplification context rather than a universal ITP mechanism. Recent platelet-surface profiling identified variable C1q, C3d, and C4d deposition with differing immunoglobulin and platelet-turnover contexts (39).

Deposition, soluble component concentration, pathway activation, and functional platelet injury are distinct levels of evidence. Platelet-bound fragments could reflect ongoing amplification, prior activation, antibody context, or increased platelet turnover. Complement may cooperate with IgG or IgM and Fc-dependent clearance, but its contribution may differ among patients and need not be independent of antibody biology.

Complement activity could resolve as immune-complex formation or antibody effector activity decreases while upstream autoreactivity persists; alternatively, deposition may remain or may be absent throughout. This variability supports complement as a research dimension, not an established subtype or clinical marker. Prospective studies should combine surface, soluble, functional, immunoglobulin, and platelet-turnover measures across defined clinical states. Any subgroup or clinical use requires prospective validation.

### 3.6 Innate immune activation

Innate immunity connects inflammatory state with antibody-dependent and antibody-independent platelet injury. Monocyte subsets and functions differ in active ITP and have changed at remission resampling, after glucocorticoid exposure, and during eltrombopag treatment (25, 40). Because these observations arise from different therapies, definitions, and intervals, they establish innate plasticity rather than durable restoration. Tissue macrophages and marrow myeloid cells may also differ from circulating monocytes.

Single-cell studies broaden the candidate innate compartment beyond conventional monocyte subsets, while spatial marrow evidence links neutrophil inflammatory signaling to megakaryocyte maturation (24, 37). The spatial mechanism is the same evidence used in the marrow section and should be understood as a cross-domain connection rather than independent replication. These findings suggest that local innate programs could influence platelet production, but discovery cell states and inferred communication require replication and functional confirmation.

Innate change after therapy may reflect direct pharmacology, altered platelet production, reduced inflammatory load, or partial immune recovery. Peripheral normalization does not establish tissue normalization. Trained immunity and inflammatory memory are relevant hypotheses, but persistence of a cytokine or surface marker is insufficient: trained immunity requires durable cell-intrinsic epigenetic or metabolic reprogramming and an altered response to secondary stimulation. Direct human ITP evidence meeting these criteria remains absent.

This distinction matters because monocytes are short-lived and can be replenished from progenitors, whereas tissue macrophage and marrow programs may persist through different cellular mechanisms. Demonstrating memory would therefore require evidence that a durable program is retained or regenerated despite turnover, together with a reproducible functional recall phenotype. Cross-sectional single-cell states cannot answer that temporal question.

Future studies should coordinate innate and adaptive measurements across active disease, platelet response, sustained treatment-free remission, and relapse; test blood-to-tissue concordance; and combine serial molecular measurements with functional recall assays. Innate trajectories may help distinguish transient inflammatory resolution from persistent tissue programs, but this distinction requires prospective validation and has no current clinical or prognostic application.

Across the six interconnected domains, autoreactive B-lineage sources, antibody-effector competence, T-cell tolerance, innate effector programs, complement amplification, and the marrow production niche may change together or asynchronously. Their overlap is biologically expected rather than a defect of the framework. The research task is to determine whether a limited and reproducible set of longitudinal measurements can distinguish transient hematologic control, treatment-related immune change, and sustained reduction in disease-relevant immune activity.

## 4 Clinical implications, limitations, and future direction

### 4.1 Clinical implications

The proposed framework currently contributes research organization and claim discipline rather than clinical implementation. It does not support automatic immune testing, longer treatment, treatment withdrawal, relapse prediction, or biomarker-guided management. Platelet response, bleeding risk, relapse pattern, treatment toxicity, comorbidities, patient preference, and treatment burden remain the basis of care (1, 2, 41). Candidate immune information could become clinically relevant only if it adds reproducible value beyond these established assessments.

Treatment-free remission is a clinically important state after tapering or discontinuation, including in studies of thrombopoietin receptor agonists (42–44). It may reflect durable disease control, but it does not by itself establish immune restoration. Conversely, a candidate immune abnormality after platelet recovery would not alone justify continued therapy.

ITP is clinically heterogeneous, and selected patients may show ANA positivity, persistent autoimmune serology, complement abnormalities, or other features associated with connective tissue disease (9–13). These findings are contextual variables rather than direct components of immune remission: ANA-positive ITP is not equivalent to SLE, and no single serological feature establishes secondary ITP, prognosis, or a treatment indication. SLE illustrates that clinical quiescence and serological activity can be dissociated, but its disease-specific targets and therapies cannot be transferred to ITP (14–17). Longitudinal assessment of clinically relevant systemic features may help characterize an autoimmune-enriched subgroup, while routine care remains anchored to platelet response, bleeding risk, relapse, toxicity, comorbidity, and patient preference.

### 4.2 Limitations

The framework integrates domains with unequal evidence maturity and heterogeneous populations, compartments, treatments, assays, comparators, and sampling times. Cross-sectional abnormalities cannot establish persistence; treatment-associated change cannot establish restoration; and clinical association cannot establish prediction. Candidate platelet-specific antibodies, systemic autoimmune features, cellular profiles, complement measures, and marrow-associated observations lack a shared operational definition or validated threshold for immune remission (7–13). Recent single-cell, spatial, repertoire, and pathway-perturbation studies strengthen mechanistic plausibility but do not establish longitudinal persistence, domain concordance, patient-level mechanism assignment, or immune remission (18–40).

Systemic autoimmune features require particular caution. ANA and related findings may enrich longitudinal risk assessment in selected patients, but ANA alone is nonspecific and does not define secondary ITP or an immune-remission state (12, 13). SLE remains a conceptual precedent only; disease-specific treatment targets, serological thresholds, and maintenance strategies cannot be transferred directly to ITP (14–17).

### 4.3 Future direction

Validation should proceed in stages. A discovery phase should use prespecified sampling during active disease, platelet response, sustained treatment-free follow-up, and relapse to identify reproducible within-patient trajectories. A derivation phase should reduce these observations to a feasible candidate signature while separating disease-related change from direct treatment effects and stable inter-individual variation. An external-validation phase should then test predefined measurements and thresholds in independent cohorts and determine whether they add information beyond platelet count, bleeding, disease duration, and treatment exposure. Clinical-utility testing should occur only if measurement and predictive validity are established.

**Table 2** supports this pathway by separating mechanistic measurements, immune-state measurements, clinical anchors, and validation endpoints. The categories specify different roles in inference: mechanistic measures test candidate pathways; immune-state measures characterize multidomain trajectories; clinical anchors define the state against which immune change is interpreted; and validation endpoints test durability, prediction, replication, and added value. They do not form a score or mandate routine testing.

## 5 Conclusion

The proposed framework reframes immune remission in ITP as a testable longitudinal construct rather than an inference from platelet recovery or a requirement for global immunological normalization. Six interconnected analytical domains organize candidate mechanisms, while a staged validation pathway links immune trajectories to durable clinical control and relapse. Platelet response and bleeding risk remain the clinical anchors. Until prespecified measurement, external validation, and added clinical value are demonstrated, immune remission should not be used as a clinical endpoint, prediction tool, or treatment rule.

## Data Availability

The original contributions presented in the study are included in the article/supplementary material. Further inquiries can be directed to the corresponding author.
